# Using graph models to find transcription factor modules: the hitting set problem and an exact algorithm

**DOI:** 10.1186/1748-7188-8-2

**Published:** 2013-01-16

**Authors:** Songjian Lu, Xinghua Lu

**Affiliations:** 1 Department of Biomedical Informatics, University of Pittsburgh, Pittsburgh, PA 15219, USA

## Abstract

Systematically perturbing a cellular system and monitoring the effects of the perturbations on gene expression provide a powerful approach to study signal transduction in gene expression systems. A critical step of revealing a signal transduction pathway regulating gene expression is to identify transcription factors transmitting signals in the system. In this paper, we address the task of identifying modules of cooperative transcription factors based on results derived from systems-biology experiments at two levels: First, a graph algorithm is developed to identify a minimum set of co-operative TFs that covers the differentially expressed genes under each systematic perturbation. Second, using a clique-finding approach, modules of TFs that tend to consistently cooperate together under various perturbations are further identified. Our results indicate that this approach is capable of identifying many known TF modules based on the individual experiment; thus we provide a novel graph-based method of identifying context-specific and highly reused TF-modules.

## Background

In order to survive, a cell responds to a variety of environmental and internal perturbations, e.g., environmental stresses and gene mutations respectively. A common response to cellular perturbations is to activate gene expression programs that induce or repress expression of genes to cope with changed homeostatus. Signals which originate as a result of the perturbation are often propagated to transcription factors (TFs), which serve as bottlenecks of signal transduction pathways that regulate transcription programs. Often multiple TFs are involved in regulating one set of genes in a cooperative manner, hence they are referred to as a TF module, and their binding sites in the genome are often referred to as cis-regulatory modules.

**Figure 1:**
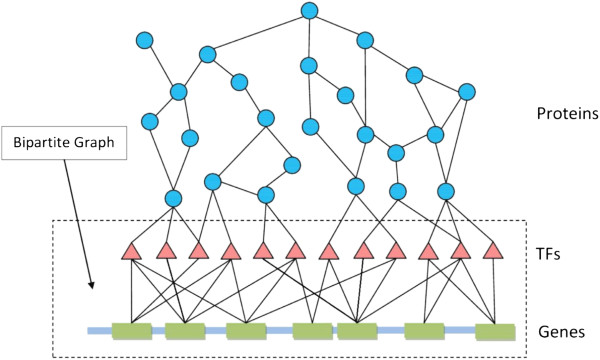
Bipartite graphs for TFs and Genes.

Figure 1 shows the concept structure of information flow, where information is transmitted through signalling proteins to TF modules that regulate gene transcriptions. The binding relations between TFs and genes can be represented by a bipartite graph.

There is an extensive body of literature on identifying cis-regulatory modules by combining a variety of data [1-7]. The most commonly used approach is to study the combinatory patterns of transcription factor binding sites (TFBSs) in a set of co-expressed genes, often derived from clustering analysis of multiple gene expression data. Since the space of combination of TFs is extraordinarily large, contemporary TF-module-searching methods usually adopt heuristic or stochastic searching algorithms, which cannot guarantee optimal solutions based on given searching criteria. In addition, searching TF modules based on clustering analysis of gene expression data introduces an implicit assumption that the enriched TFBSs regulate the expression of the genes under all conditions. However, in reality, activation of TFs and subsequent binding to their TFBSs are often dependent on the state of cellular signaling systems in a context-specific fashion. Therefore, there is a need for identification methods that are capable of identifying an optimal combination of TFs that explain gene expression patterns in a context-specific setting, which need cannot be effectively addressed within the conventional statistical framework. Therefore, in this study, we address the task of identifying cooperative TFs from a new perspective—using a graph-based approach.

The main ideas underlying our approaches are as follows: First, given a set of differentially expressed genes identified from an individual microarray experiment, we identify the cooperative TFs by searching for a minimum set of TFs that bind (cover) these genes with high reliabilities (reflected as weights associated with TFs) and where each gene is covered by at least *t* TFs, a weighted *t*-cover hitting set problem. Our results indicate that the latter constraint enables our approach to recover known cooperative TFs. Second, based on sets of TFs identified from each microarray experiment, we further find a set of TFs that tend to cooperatively function together in multiple instances, thus revealing TF modules at the systems level.

The *t*-cover hitting set problem is an NP-hard problem. As the previous best algorithm for solving this problem cannot deal with the weighted case and has impractical time complexity for our task, we developed a new *exact* algorithm, which not only deals with the weighted case of the problem, but also has significantly less time complexity, thus enabling us to find exact solutions for the problems in our study.

## Problem Formulation

### Data sets

• We collected the gene expression data from the seminal study by Hughes *et al.,*[8], in which transcriptional responses to systematic genetic and pharmacological perturbations were investigated.

• We collected a protein-DNA interaction graph from Huang *et al.,* and Yeger-Lotem *et al.,*[9,10]. In this bipartite graph, vertices on one side are TF proteins while the vertices on the other side are potential target genes. An edge between a TF and a gene indicates that the TF likely binds to the promoter of the gene as based on the ChIP-chip experimental results [9,10], and the edge weight reflects the reliability of a binding event between a corresponding TF and gene pair.

### Finding cooperative TFs for a set of co-regulated genes

Given a set of co-regulated genes, finding a set of cooperative TFs regulating them is a challenging problem in studying transcriptional regulation [2]. In this study, we cast the task as a graph problem, referred to as the weighted *t*-cover hitting set problem, and designed an efficient algorithm to solve it.

For a set of co-regulated genes, we induce a subgraph from the bipartite graph representing protein-DNA interaction. The new graph remains a bipartite graph, with one part being all TFs and the other being the given co-regulated genes. If a TF is connected to a gene, we say that the TF covers the gene. The task is to find a subset of TFs with minimum weight, called the *t*-TF cover, such that each gene is covered by at least *t* TFs in the *cover*. The weight of the *t*-TF cover is the sum of the weights of TFs in the cover. We defined the weight of a TF to be the reciprocal of the sum of edge weights, which is defined in the input bipartite graph by Huang *et al.,*[9,10]. More specifically, if TF *t*_1_ is connected to genes *g*_1_, *g*_2_, …, *g*_*k*_, then $\text{weight}(t_{1})=\frac{1}{\overset{k}{\underset{i=1}{\sum }}\text{Edge}\text{\_}\text{Weight}(t_{1},g_{i})}$. Recall that, in our case, an edge weight reflects the binding reliability between the TF and its target gene; as such, a TF which interacts with many genes with high reliability will have a small TF weight, and thus is more likely to be included in the solution of the *t*-TF cover problem.

When *t* = 1, the solution finds a set of TFs such that each gene must be covered by at least one TF, which will return a minimum set of TFs that covers all those co-regulated genes. Such a result is not interesting because we aim to find cooperative TFs. By constraining *t* to be greater than 1, we are able to try to find a set of TFs such that each gene must be covered by more than one TF in the set. By definition, a solution will preferentially search for and include TFs that co-cover as many genes in a target set as possible and with as high reliability as possible. From a biological viewpoint, the more genes a set of TFs co-covers, the more likely the TFs act cooperatively to regulate the genes. *Thus, finding an optimal solution for the t-TF cover problem in this setting is a biologically sensible approach for identifying TF modules.*

The problem of finding the *t*-TF cover is NP-hard, where the problem is equivalent to two well-known NP-hard problems, the set multicover problem and the *t*-cover hitting set problem [11,12]. The *t*-TF cover problem can be reduced to the *t*-cover hitting set problem easily. The formal definition of the weighted *t*-cover hitting set is as follows: 

 weighted *t*-cover hitting set: Given a universal set *X* of *m* elements, a weight function *w*(*x*) : *X* → *R*^+^, a family $T=\{S_{1},\ldots ,S_{n}\}$ of subsets of *X*, where the size of any subset in T is at least *t*, and an integer *t*, find a subset *H* ⊆ *X* of minimum weight such that every subset in T has at least *t* elements in *H*, where the weight of *H* is defined as $\underset{x\in H}{\sum }w(x)$. We denote an instance of the problem as (X,T,w,t) and call *H* the minimum *t*-cover hitting set.^a^

As mentioned above, the weighted *t*-cover hitting set problem is NP-hard and the previous best algorithm for the unweighted case of the problem has a time complexity of *O*((*t* + 1)^*n*^*nm*) [12]. In terms of our application, *n* is the number of co-regulated genes and *m* is the number of TFs; these two numbers are usually large enough to render the existing algorithms impractical for our problem. Thus, designing an efficient exact algorithm to solve the weighted *t*-cover hitting set problem becomes the main goal and therefore the main contribution of this study.

### Identify repeatedly used TF modules

For each perturbation instance, a set of differentially expressed genes (considered to be co-regulated genes) can be decided. Using the method in the previous subsection, we can obtain a set of cooperative TFs for each perturbation instance. At this stage, a biologist may want to find modules of TFs that are repeatedly used under various experimental conditions, as they are more likely to be part of signal transduction pathways that are repeatedly involved in responses to different perturbations.

We refer to a set of TFs sharing a common set of target genes as a “hard” TF clique. The formal definition of a “hard” TF clique is as follows: a subset of TFs *T* forms a “hard” TF clique if there exists a subset of genes *G* such that *T* and *G* make a complete induced subgraph in the protein-DNA interaction graph. From a biological viewpoint, the more common target genes are shared by the TFs, the more likely the TFs truly cooperate to regulate the genes. Thus, we weigh a hard clique using the number of common target genes covered by the TFs to reflect the “goodness” of the clique. In such a setting, one way to find the TF modules that are repeatedly used in many perturbation instances is to find hard TF cliques that occur in multiple instances, where the overall weight of such hard cliques is the sum of weights of the clique from all instances. The higher the weight of a clique, the more likely the TFs in the clique will function as a module. However, as there are usually noise and errors in microarray data, it is difficult to consistently find hard cliques from multiple instances. To address this issue, we introduce a new formulation: search for a TF module whose member TFs tend to cooperatively regulate gene expression in multiple instances but not necessarily in all perturbation instances.

First, we use the cooperative TFs found in each instance to make one TF-TF-relation graph for each case of *t* = 1, 2, 3, 4. In such a graph, each TF is a node; a weighted edge between a pair of TFs is added if the TFs are found to cooperate in one or more instances, of which the weight is the sum of the number of common target genes from all instances. Hence, the higher the weight of an edge, the more cooperation instances the two TFs have. Then we search for all 3-cliques and 4-cliques from such a TF-TF graph and sort them by weights, where the weight of a clique is the sum of all edge weights for the clique. Since we have relaxed the requirement so that the TFs in these cliques do not need to form a hard clique in all individual instances, we refer to this approach as finding “soft” TF cliques. At this stage, we limit the search for cliques to 3-cliques and 4-cliques because our data [9,10] indicated that only 15% of genes in yeast are connected to more than 5 TFs.

The clique finding problem is a well-known NP-hard problem. However, since we only need to find cliques of size 3 and size 4, we can search for cliques of these sizes in a graph with at most 214 (the total number of TFs in our case) vertices in a reasonable time. In the Results section, we will compare the results for “soft” 3-cliques for the cases of *t* = 1, 2, 3, 4 and hard 3-cliques for the case of *t* = 3, and “soft” 4-cliques for the cases of *t* = 1, 2, 3, 4 and hard 4-cliques for the case of *t* = 4.

## Exact algorithm for *t*-cover problem

**Figure 2:**
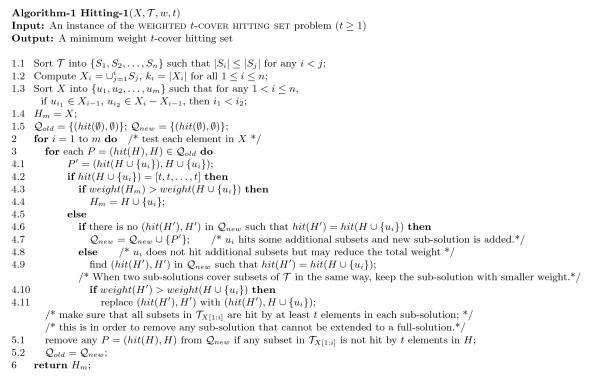
**Algorithm for solving the weighted*t*-cover hitting set problem.**

Usually, finding an exact optimal solution for an NP-hard problem in practical time is difficult; it is not uncommon for such a program to run as long as months or years. Hence, heuristic or greedy algorithms are often designed to approximately solve NP-hard problems. However, heuristic or greedy algorithms cannot guarantee the performance. Therefore, in order to design an exact algorithm, we investigated the characteristic of our problem and found that the degrees of many genes were very small (i.e., they were only connected to a small number of TFs in the protein-DNA interaction graph [9,10]). More specifically, about 70% of genes had degrees less than or equal to 3, about 85% of genes had degrees which are at most 5, and about 96% of genes had degrees less than or equal to 10. This characteristic enabled us to design an efficient exact algorithm, which was presented in Figure 2, for the problem. In addition to its efficiency, our algorithm is the first exact algorithm that can solve a weighted *t*-cover hitting set problem.

Before proving the correctness and time complexity of the algorithm, we give the basic idea of our algorithm, which is based on the dynamical programming technique. When we expand the sub-solutions, if two sub-solutions *H*_1_ and *H*_2_ hit exactly the same group of subsets in T, we prove that keeping any one of these two sub-solutions is sufficient. Hence, if |T|=n, then we keep at most (*t* + 1)^*n*^ different sub-solutions (Note: there are *n* subsets, where each subset can be hit by 0, 1, 2, …, or at least *t* elements in the sub-solution. Hence, totally, there are at most (*t* + 1)^*n*^ cases.). This is the main part of the time complexity and the space complexity. In the algorithms, we also sort T such that sizes of subsets in T are ordered from the smallest to the largest (when there is a tie, an arbitrary order suffices). If sizes of many subsets in T are bounded, such as sizes of first *k* subsets {*S*_1_,*S*_2_,…,*S*_*k*_} are bounded by *d*, we also sort *X* such that the first $\left|\cup_{i=1}^{\;j}dS_{i}\right|$ elements are $\cup_{i=1}^{\;j}S_{i}$ for all *j* = 1, 2, …, *k*. Hence, the first $\left|\cup_{i=1}^{k}S_{i}\right|$ elements are $\cup_{i=1}^{k}S_{i}$. In the algorithm, we add the elements of sorted *X* orderly into the sub-solutions (i.e., first, try to add the first element of *X* into sub-solutions. Then try to add the second element of *X* into sub-solutions, and so on.) such that, first, we make *S*_1_ be hit by at least *t* elements of each sub-solution. Then we make *S*_1_ and *S*_2_ be hit by at least *t* elements of each sub-solution, and so on. It is easy to know that when we have considered first $\left|\cup_{i=1}^{k}S_{i}\right|$ elements in the sorted *X*, all {*S*_1_,*S*_2_,…,*S*_*k*_} are hit by at least *t* elements of each sub-solution. At that time, the number of sub-solutions is bounded by $2^{\left|\cup_{i=1}^{k}S_{i}\right|}$ (all possible combinations of first $\left|\cup_{i=1}^{k}S_{i}\right|$ elements in the sorted *X*). After that, as we only need to remember the hitting statuses of remaining *n*-*k* subsets in T, the number of sub-solutions is bounded by (*t* + 1)^*n*-*k*^. We will show that if sizes of many subsets in T are bounded, $2^{\left|\cup_{i=1}^{k}S_{i}\right|}$ and (*t* + 1)^*n*-*k*^ will be much smaller than (*t* + 1)^*n*^.

Let *X* = {*u*_1_, *u*_2_, …, *u*_*m*_} and $T=\{S_{1},S_{2},\ldots ,S_{n}\}$. We define $T_{X[1:i]}=\{S|S\in T\text{and}\;S\subset \{u_{1},u_{2},\ldots ,u_{i}\}\}$ for 1 ≤ *i* ≤ *m*. Let *H* be a subset of *X*. We define *hit*(*H*) = [*c*_1_, *c*_2_, …, *c*_*n*_], and $\text{weight}(H)=\underset{u\in H}{\sum }w(u)$, where *c*_*i*_ = *hit*(*H*)[*S*_*i*_] = *min*(*t*,|*S*_*i*_∩*H*|) for 1 ≤ *i* ≤ *n*, i.e., *c*_*i*_ remembers how many element(s) in *S*_*i*_ is(are) in *H* (Note: if any *S*_*i*_ already has at least *t* elements in a partial solution, we can removed *S*_*i*_ from the problem and do not need to further remember its covering status. Hence, there is no need to remember any |*S*_*i*_∩*H*| that is large than *t*). Following lemmas are needed in the proof of the main theorem.

### Lemma 0.1

Let *H*_1_, *H*_2_, *H*^′^ be three subsets of *X* such that *H*_1_ ∩ *H*^′^ = *∅* and *H*_2_ ∩ *H*^′^ = *∅*. If *hit*(*H*_1_) = *hit*(*H*_2_), then *hit*(*H*_1_ ∪ *H*^′^) = *hit*(*H*_2_ ∪ *H*^′^).

### Proof

As *hit*(*H*_1_) = *hit*(*H*_2_), for any $S_{i}\in T$, *hit*(*H*_1_)[*S*_*i*_] = *hit*(*H*_2_)[*S*_*i*_], i.e., *min*(*t*, |*S*_*i*_∩*H*_1_|) = *min*(*t*, |*S*_*i*_ ∩ *H*_2_|). Furthermore, because *H*_1_ ∩ *H*^′^ = *∅* and *H*_2_ ∩ *H*^′^ = *∅*, we will have that, for any $S_{i}\in T$, *min*(*t*, |*S*_*i*_ ∩ (*H*_1_ ∪ *H*^′^)|) = *min*(*t*, |*S*_*i*_∩*H*_1_| + |*S*_*i*_ ∩ *H*^′^|) = *min*(*t*, |*S*_*i*_ ∩ *H*_2_| + |*S*_*i*_∩*H*^′^|) = *min*(*t*, |*S*_*i*_∩(*H*_2_∪*H*^′^)|). Therefore, *hit*(*H*_1_∪*H*^′^) = *hit*(*H*_2_∪*H*^′^) and the lemma is proved. □

The Lemma 0.1 guarantees that if any two sub-solutions cover in the same way, then keeping the sub-solution with the smaller weight is enough.

### Lemma 0.2

Let $H^{\ell }=\{u_{i_{1}},u_{i_{2}},\ldots ,u_{i_{\ell }}\}$, whose elements are in the same order as in the sorted *X* in **Algorithm-1** (i.e., if *j*_1_ < *j*_2_ with respect to the index of *H*^*ℓ*^, then $i_{j_{1}}<i_{j_{2}}$ with respect to the index of *X*), be the minimum *t*-cover hitting set, and $H_{j}^{\ell }=\{u_{i_{1}},u_{i_{2}},\ldots ,u_{i_{j}}\}$, 1 ≤ *j* ≤ *ℓ*. For any 1 ≤ *j* ≤ *ℓ*, if there is a $H\subset \{u_{1},u_{2},\ldots ,u_{i_{j}}\}$ such that $\text{hit}(H)=\text{hit}(H_{j}^{\ell })$, then $\text{weight}(H)\geq \text{weight}(H_{j}^{\ell })$.

### Proof

Let $H^{\prime }=\{u_{i_{j+1}},u_{i_{j+2}},\ldots ,u_{i_{\ell }}\}$. Then *H* ∩ *H*^′^ = *∅*. If $\text{weight}(H)<\text{weight}(H_{j}^{\ell })$, then by Lemma 0.1, *H* ∪ *H*^′^ will be a *t*-cover hitting set with a smaller weight than the weight of *H*^*ℓ*^, which causes contradiction. Hence, the lemma is correct. □

The Lemma 0.2 shows that **Algorithm-1** always keeps a sub-solution that will lead to the full-solution with minimum weight. Now, let us present and prove the main theorem.

### Theorem 0.3

The weighted *t*-cover hitting set problem can be solved in *O*((*t* + 1)^*n*^*mnt*) time and in *O*((*t* + 1)^*n*^*nt*) space, where *m* is the size of the ground set and *n* is the number of subsets for the given instance. If, furthermore, the problem has at least $\frac{n}{1+d/\underset{2}{\log}(t+1)}$ subsets whose sizes are upper bounded by *d*, then the problem can be solved in $O({\left({\left(t+1\right)}^{d/(d+\underset{2}{\log}(t+1))}\right)}^{n}\text{mnt})$ time and in $O({\left({\left(t+1\right)}^{d/(d+\underset{2}{\log}(t+1))}\right)}^{n}\text{nt})$ space.

### Proof

We first prove the *correctness* of the algorithm.

Given an instance (X,T,w,t) of the weighted *t*-cover hitting set problem, let *X* = {*u*_1_, *u*_2_, …, *u*_*m*_}, where *X* is sorted as shown in **Algorithm-1** such that the order of elements in *X* is as *S*_1_, *S*_2_ - *X*_1_, …, *S*_*n*_-*X*_*n*-1_, where $X_{j}\;=\;\cup_{i=1}^{\;j}S_{i}$. Let $H^{\ell }\;=\;\{u_{i_{1}},u_{i_{2}},\ldots ,u_{i_{\ell }}\}$, whose elements are in the same order as in the sorted *X* (i.e., if *j*_1_ < *j*_2_ with respect to the index of *H*^*ℓ*^, then $i_{j_{1}}\;<\;i_{j_{2}}$ with respect to the index of *X*), be the minimum *t*-cover hitting set. Let $H_{j}^{\ell }\;=\;\{u_{i_{1}},u_{i_{2}},\ldots ,u_{i_{j}}\}$ for all 0 ≤ *j* ≤ *ℓ*, where $H_{0}^{\ell }\;=\;\emptyset$.

To prove correctness, we **claim** that when the for loop in step 2 of **Algorithm-1** is at loop *i* = *i*_*j*_ for all 1 ≤ *j* ≤ *ℓ* (Note: $u_{i_{j}}$ is the *j*th element in *H*^*ℓ*^ and *i*_*j*_ th element in *X*), there exists a *P* = (*hit*(*H*), *H*) in $Q_{\text{old}}$ (loop in step 3) such that $\text{hit}(H)=\text{hit}(H_{j-1}^{\ell })$ and $\text{weight}(H)=\text{weight}(H_{j-1}^{\ell })$. We prove this claim by mathematical induction on *j*. 

• **Induction basis**. In the case of *j* = 1, as for any *i* < *i*_1_, $T_{X[1:i]}=\emptyset$ (else, *H*^*ℓ*^ cannot be the solution), no sub-solution will be removed in step 5.1 for all loops of *i* < *i*_1_ in step 2. Hence, when *i* = *i*_1_, *P* = (*hit*(*∅*), *∅*) is in $Q_{\text{old}}$. The claim is correct.

• **Induction step**. Suppose that when *j* < *q* ≤ *ℓ*, the claim is true. Hence, when *i* = *i*_*q*-1_ in the loop of step 2, there exists a *P* = (*hit*(*H*), *H*) in $Q_{\text{old}}$ such that $\text{hit}(H)=\text{hit}(H_{q-2}^{\ell })$ and $\text{weight}(H)=\text{weight}(H_{q-2}^{\ell })$. Then by Lemma 0.1, $\text{hit}(H\cup \{u_{i_{q-1}}\})=\text{hit}(H_{q-2}^{\ell }\cup \{u_{i_{q-1}}\})=\text{hit}(H_{q-1}^{\ell })$, and $\text{weight}(H\cup \{u_{i_{q-1}}\})=\text{weight}(H_{q-1}^{\ell })$. Therefore, $P^{\ell }=(\text{hit}(H_{q-1}^{\ell }),H_{q-1}^{\ell })$ will be saved into $Q_{\text{new}}$ unless there is another *P*^′^ = (*hit*(*H*^′^), *H*^′^) in $Q_{\text{new}}$ such that $\text{hit}(H^{\prime })=\text{hit}(H_{q-1}^{\ell })$, and $\text{weight}(H^{\prime })=\text{weight}(H_{q-1}^{\ell })$. By Lemma 0.2, if any *P*^′^ = (*hti*(*H*^′^), *H*^′^) such that $\text{hit}(H^{\prime })=\text{hit}(H_{q-1}^{\ell })$, and $\text{weight}(H^{\prime })=\text{weight}(H_{q-1}^{\ell })$ is already saved into $Q_{\text{new}}$, it will not be replaced. Furthermore, as any $S\in T_{X[1:i]}$ for *i* < *i*_*q*_, *hit*(*H*^′^)[*S*] = *t* (otherwise, it would cause a contradiction that *H*^*ℓ*^ is a solution as no element in $\left\{u_{i_{q}},\ldots ,u_{i_{\ell }}\right\}$ will cover *S*). Hence, *P*^′^ = (*hit*(*H*^′^), *H*^′^) will not be removed when loop *i* < *i*_*q*_ in loop 2. Thus, *P*^′^ = (*hit*(*H*^′^), *H*^′^) will be in $Q_{\text{old}}$ when *i*=*i*_*q*_ in the loop of step 2, i.e., the claim is still true when *j* = *q*.

Therefore, when *j* = *ℓ*, we will save a (*hit*(*H*), *H*) into $Q_{\text{new}}$ such that $\text{hit}(H)=\text{hit}(H_{\ell }^{\ell })=\text{hit}(H^{\ell })$, and *weight*(*H*) = *weight*(*H*^*ℓ*^), i.e., we will find the minimum *t*-cover hitting set. The correctness of **Algorithm-1** is proved.

Next, we consider the time complexity and space complexity of the algorithm. Step 2 loops |*X*| = *m* times. Step 3 loops $\left|Q_{\text{old}}\right|$ times. As $Q_{\text{old}}$ only remember different combinations of [*c*_1_, *c*_3_, …, *c*_*n*_] and each *c*_*i*_ is between 0 and *t*, it is obvious that $|Q_{\text{old}}|\leq {\left(t+1\right)}^{n}$. Steps 4.1 to 4.4 take *O*(*nt*) time. Steps 4.6 to 4.11 can be finished in *O*(log2(*t* + 1)^*n*^) = *O*(*n* log2(*t* + 1)) time if we use AVL tree to implement $Q_{\text{new}}$ and $Q_{\text{old}}$. Hence the total time complexity is *O*((*t* + 1)^*n*^*mnt*).

In the case that T has at least $\frac{n}{1+d/\underset{2}{\log}(t+1)}$ subsets whose sizes are bounded from above by *d*, then when $i=\frac{\text{dn}}{1+d/\underset{2}{\log}(t+1)}$, both $Q_{\text{old}}$ and $Q_{\text{new}}$ have at most $2^{\frac{\text{dn}}{1+d/\underset{2}{\log}(t+1)}}={\left({\left(t+1\right)}^{\frac{1}{1+\underset{2}{\log}(t+1)/d}}\right)}^{n}$ elements. Furthermore, when $i=\frac{\text{dn}}{1+d/\underset{2}{\log}(t+1)}$, for any *P* = (*hit*(*H*), *H*) in $Q_{\text{old}}$ or in $Q_{\text{new}}$, if let *hit*(*H*) = [*c*_1_, *c*_2_, …, *c*_*n*_], then *c*_*j*_ = *t* for $1\leq j\leq \frac{n}{1+d/\underset{2}{\log}(t+1)}$. Hence, when $i>\frac{\text{dn}}{1+d/\underset{2}{\log}(t+1)}$, all elements in $Q_{\text{old}}$ or in $Q_{\text{new}}$ have at most ${\left(t+1\right)}^{n-\frac{n}{1+d/\underset{2}{\log}(t+1)}}$ combinations of *hit*(*H*), i.e., the size of $Q_{\text{old}}$ or $Q_{\text{new}}$ is always bounded from above by ${\left(t+1\right)}^{n-\frac{n}{1+d/\underset{2}{\log}(t+1)}}={\left({\left(t+1\right)}^{\frac{1}{1+\underset{2}{\log}(t+1)/d}}\right)}^{n}$. Therefore, the total time complexity is $O({\left({\left(t+1\right)}^{\frac{1}{1+\underset{2}{\log}(t+1)/d}}\right)}^{n}\text{mnt})$.

It is obvious that the space complexity is $O(|Q_{\text{old}}|\cdot \max$ (lengthes of elements in $Q_{\text{old}}))=O(|Q_{\text{new}}|)\cdot \max$ (lengthes of elements in $Q_{\text{new}}))$. The lengthes of elements in both $Q_{\text{old}}$ and $Q_{\text{new}}$ are bounded from above by *O*(*nt*). Therefore, in the general case, the space complexity is *O*((*t* + 1)^*n*^*nt*) and in the case sizes of many subsets in T are bounded from above by *d*, the space complexity is $O({\left({\left(t+1\right)}^{d/(d+\underset{2}{\log}(t+1))}\right)}^{n}\text{nt})$. □

The **Algorithn-1** only reports one solution with the minimum weight, even problems in application have multiple solutions with the minimum weight. The setting of weights of TFs increases the probability that any solution with the minimum weight includes most correct TFs that regulate differently expressed genes. However, in some cases of the application, we may also want to study other top weight solutions (as the data error, the actual solution may not have the minimum weight). By modifying the algorithm such that for each distinct cover way, save *k* top weight sub-solutions, then the new algorithm can output *k* top weight solutions. It is also easy to prove that the time complexity and space complexity of the new algorithm will only increase by a ratio *k*.

Before we finish this section, we briefly summarize the time complexity of our algorithm and compare it with the previously reported one [12]: 

• If there are at least $\frac{n}{1+d/\underset{2}{\log}(t+1)}$ subsets whose sizes are upper bounded by *d*, then the time complexity of our algorithm is $O({\left({\left(t+1\right)}^{d/(d+\underset{2}{\log}(t+1))}\right)}^{n}\text{mnt})$, while the time complexity of the previous best algorithm is always *Ω*((*t* + 1)^*n*^*mn*) [12] (and only works for the unweighted case). As *d*/(*d* + log2(*t* + 1)) < 1, ${\left({\left(t+1\right)}^{d/(d+\underset{2}{\log}(t+1))}\right)}^{n}$ is much less than (*t*+1)^*n*^. For example, if we let *d* = 5 (note: 85% of genes in our case have degrees less than or equal to 5) and *t* = 2, 3, 4, our algorithm is bounded by *O*(2.303^*n*^*mn*), *O*(2.692^*n*^*mn*), or *O*(3.002^*n*^*mn*) respectively, while the the previous best algorithm is bounded by *Ω*(3^*n*^*mn*), *Ω*(4^*n*^*mn*), or *Ω*(5^*n*^*mn*) respectively. Suppose *n* = 30, then our algorithm is at least 1393 times faster if *t* = 2, or 48131 times faster if *t* = 3, or 1108459 times faster if *t* = 4 than the previous best algorithm.

• The time complexity shown above is only the worst case upper bound; in most cases, the actual time complexity is usually much better. In fact, we can further improve the running time by removing a gene from the graph whenever we find a gene’s degree is less than *t*. Thus the value of *n* can be greatly reduced.

## Results

When applied to the protein-DNA interaction graph induced from ChIP-chip experiments [9,10] and the results from microarray experiments from Hughes *et al.,*[8], our algorithms identify TF modules at two levels: First, given genes differentially expressed in each perturbation experiment, we find a set of cooperative TFs at the perturbation-instance level in a context-specific manner. Second, we further combine context-specific TFs to find TF modules that are repeatedly utilized at the system level. In the following subsections, we show that our approach produces biologically sensible results at both levels.

### TFs for sets of co-regulated genes

For each yeast genetic/pharmacological perturbation experiment, we identified genes that were differently expressed. Then, we applied our weighted *t*-cover hitting set algorithm to each bipartite component induced by connecting the differentially expressed genes and TFs to identify a set of cooperative TFs from the component. In addition, we applied our algorithm by setting *t* to 2, 3, and 4 respectively to investigate the impact of setting this parameter. Empirically, we found that setting *t* = 2 was sufficient to force our algorithm to find a set of cooperative TFs. We recommend setting *t*=2 as a beginning parameter and exploring other settings based on the degree of the genes in the organism of interest. After inspecting the resulting TF modules, we found many of them to be already well-known. Due to page limitations, we will discuss just one of the well-known modules identified by our algorithm below.

The pheromone response pathway of *S. cerevisiae*, which consists of more than 20 proteins [13], is a well-studied signal transduction pathway in yeast. When this pathway is activated by pheromone, a well-studied transcription program is initiated which is known to be cooperatively regulated by TFs: *Ste*12*p*, *Dig*1*p*/*Dig*2*p*[13] or *Ste*12*p*, *Dig*1*p*/*Dig*2*p*, and *Mcm*1*p*[9]. In the experiments by Hughes *et al.,* 12 gene-encoding proteins in the pathway were perturbed, and many canonical pheromone response genes were differentially expressed. Hence, one may expect that *Ste*12*p*, *Dig*1*p* or *Ste*12*p*, *Dig*1*p*, and *Mcm*1*p* are involved in mediating these responses. Indeed, our results show that, when we set *t* = 2, *Ste*12*p*, *Dig*1*p* were returned as members of the set of cooperative TFs identified in all those 12 perturbations and *Ste*12*p*, *Dig*1*p*, *Mcm*1*p* were found in the set of cooperative TFs in 9 out of those 12 perturbation experiments. In comparison, when we set *t* = 1, *Ste*12*p*, *Dig*1*p* were found in 5 instances, and *Ste*12*p*, *Dig*1*p*, *Mcm*1*p* were found in only 1 instance. These results indicate that our algorithm is capable of identifying cooperative TFs from individual experiments in a context-specific manner.

**Figure 3:**
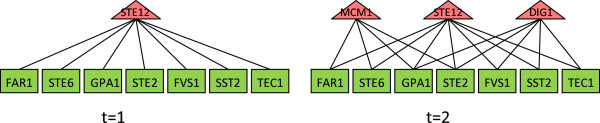
**Comparing TF module for *t = 1* and *t = 2*.**

We further studied the coverage of 7 *robust* pheromone response genes (i.e., their expression levels change significantly in almost all 12 perturbations of genes on the pheromone response pathway), namely *FAR*1, *FUS*1, *GPA*1, *SST*2, *STE*2, *STE*6, *TEC*1, to investigate how they were covered by TFs in the results obtained from the *t*-cover hitting set algorithm. Figure 3 shows the coverage of these genes by the TFs in the results returned by *t*-TF cover algorithm. As expected, when *t* = 1, the algorithm failed to find cooperative TFs but returned *Ste*13*p* as the regulating TF. On the other hand, when we set *t* = 2, the algorithm returned all three TFs, *Ste*12*p*, *Dig*1*p*, and *Mcm*1*p*, as the members of solution TFs covering these genes, which form a dense graph.

Above examples show that if we know a set of genes that are co-regulated, our new program can find correct TFs that regulate them when we set *t* to 2 or 3. There exist more examples to indicate that the algorithm is working as expected.

### Finding TF modules involved in multiple instances

Using the *t*-cover hitting set results (for *t* = 1, 2, 3, 4) from 300 microarray data, we constructed a TF-TF relation graph and searched for 3-cliques and 4-cliques in the graph. The cliques are ranked according to their weights; the larger the weight, the higher the rank is. Our assumption is that, because high-ranking cliques consist of the TFs deemed to function cooperatively in multiple instances by our algorithm, they may truly function as partners in real biological settings. We evaluated this assumption looking at two factors: 1) whether members of the cliques are known to function as a module based on literature, and 2) whether the members of the cliques have shown physiological or genetic interactions in previous experiments.

**1. High scores for known cooperative TFs:** We first inspected the top 20 high-ranking 3 cliques identified in the TF-TF graph derived from the results of the *t*-cover hitting set with *t*=2. We found that the majority of them are well known cooperative TF modules. For example, TFs participating in cell cycle checkpoint [9], *Swi*4*p*, *Swi*6*p*, and *Mbp*1*p*, ranked 2nd and, interestingly, the 3-clique including *Dig*1*p*, *Mcm*1*p*, and *Ste*12*p* (the pheromone responding TF module) ranked 4th. Similarly, many of the high ranking 4-cliques constitute TF modules that are also supported by existing knowledge. However, it is interesting to note that the 3-clique that ranked the highest consists of *Dig*1*p*, *Ste*12*p* and *Tec*1*p*, for which we could not find any published results reporting that they work together; thus it would be an interesting case to investigate if our approach has found previously unknown interactions.

We further investigated if the input data, the TF modules returned using the *t*-cover hitting sets with different settings of *t*, has an impact on the results of TF cliques. We found that the ranking and the members of cliques were similar when *t* was set to 2, 3, and 4, respectively. However, the results for when *t* = 1 and for hard cliques were totally different. For example, when the TF-TF graph is built using the results with *t* = 1, the first 3-clique containing *Swi*4*p*, *Swi*6*p*, and *Mbp*1*p* ranks 254th. Furthermore, if we rank this commonly used TF module by finding common “hard” cliques, the highest ranking 3-clique containing *Dig*1*p*, *Mcm*1*p*, and *Ste*12*p* ranks 273rd. Thus, the results indicate that, by setting *t* > 1 and finding “soft” cliques, our methods enhance the capability of finding TF modules that are repeatedly utilized in multiple conditions, and are thus likely to play key roles in cellular signal transduction systems. Due to page limitation, we made the results available by listing the top 100 soft 3-cliques and 4-cliques on the supplement.

**2. Interactions between proteins inside the cliques:** From a biological point of view, if a set of TFs work together to regulate the transcription processes, there should be physical or genetic interactions among those TFs. If the results of the *t*-cover hitting set algorithm truly capture the cooperations, one would expect that TFs in the repeatedly utilized cliques have a high probability of interacting. The following section evaluates the TF cliques through analyzing their protein-protein interactions, where we define that there exists an interaction between a pair of TFs if they have a physical interaction, a genetic interaction determined by synthetic lethality [14], or both.

**Figure 4:**
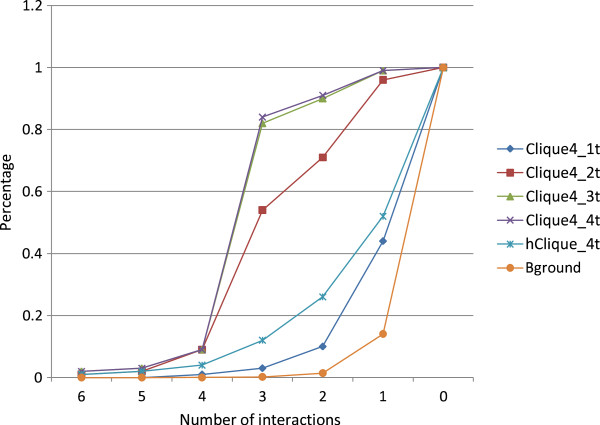
**Comparing interaction rate of cliques for different methods.** (Note: a point (3,0.54) on the curve means that 54% of top 100 score cliques for the corresponding method have at least 3 interactions).

We constructed multiple TF-TF relation graphs using the results from the *t*-cover hitting set algorithm with *t* = 1, 2, 3, 4 to assess the impact of setting *t*. From each graph, we identified the top 100 4-cliques and assessed the percent (aka, the probability) that the cliques have at least a given number of interactions. As a control, we also randomly sampled 100 groups of 4 TFs for comparison. The results are plotted as a cumulated distribution in Figure 4. From the figure, we can see that the majority of randomly picked TF groups have no interaction, with less than 20% of groups containing 1 or more interaction(s). Similarly, the curve for the cliques that are derived from the results with *t* = 1 —a setting that is not optimized for finding cooperations among TFs—is located close to that curve of the random groups. On the other hand, with *t* = 2, 3, 4, over 50% of 4-cliques contain at least 3 interactions. These results indicate that when one sets *t* > 1, the *t*-cover hitting set algorithm indeed strives to find the TFs that cooperatively regulate transcriptions; the results also indicate that setting *t* = 2 is sufficient to find cooperative TFs.

To illustrate the difference in performance between the “soft” and “hard” cliques, we also identified the top 100 “hard” 4-cliques that were obtained directly from the *t*-cover hitting set for individual perturbation instances by setting *t* = 4 and studied interactions among the TFs in these cliques. The results show that the performance of the “hard” cliques is superior to those of the random TF groups and the “soft” cliques derived from *t* = 1 results, indicating that the *t*-cover hitting set algorithm is capable of revealing cooperation among TFs. However, “hard” cliques consistently underperform in comparison with the “soft” cliques that are derived from integrating results in multiple instances (i.e., except the case of *t* = 1), which indicates that the information integration approach further enhances the quality of the TF modules.

## Conclusion

In this paper, we have developed graph-based approaches to address the problem of finding cooperative TF modules at two levels. First, given a set of co-regulated genes, we find a set of TFs that cooperatively regulate the genes in a context-specific manner. Second, given a collection of context-specific TF modules, we find the TFs that tend to function cooperatively in multiple instances at systems level, where the behind idea here is: if two TFs are working together in multiple time, then it is more possible that they are in the same TF module. For the first part, we cast the task as a weighted *t*-cover hitting set problem and developed an exact algorithm to solve the problem. The main contribution of this paper is that, by taking advantage of the knowledge of the limited gene degrees, we have developed a very efficient exact algorithm capable of solving the problem at hand in a practical time. For the second problem, we cast the task as a clique-finding problem, and our approach produced results that are biologically sensible and generate new biological hypotheses. Our graph-based approaches are significantly different from statistics-based approaches, hence providing a new perspective to study transcriptional regulation [2].

## Endnote

^a^In applications, if there is any subset whose size is less than *t*, we add dummy element/elements to make its size to *t*.

## Competing interests

The authors declare that they have no competing interests.

## Authors’ contributions

Both SL and XL analyzed biological data, established and modified the mathematical model, and drafted the manuscript. SL made the major contribution in designing the algorithm. Both authors read and approved the final manuscript.
